# The Protective Role of E-64d in Hippocampal Excitotoxic Neuronal Injury Induced by Glutamate in HT22 Hippocampal Neuronal Cells

**DOI:** 10.1155/2021/7174287

**Published:** 2021-10-20

**Authors:** RuiJin Xie, TianXiao Li, XinYu Qiao, HuiYa Mei, GuoQin Hu, LongFei Li, Chenyu Sun, Ce Cheng, Yin Cui, Ni Hong, Yueying Liu

**Affiliations:** ^1^Affiliated Hospital of Jiangnan University, No. 1000, Hefeng Avenue, Wuxi 214122, China; ^2^Wuxi School of Medicine, Jiangnan University, Wuxi, China; ^3^AMITA Health Saint Joseph Hospital Chicago, 2900 N. Lake Shore Drive, Chicago, 60657 Illinois, USA; ^4^The University of Arizona College of Medicine at South Campus, 2800E. Ajo Way, Tucson, AZ, USA; ^5^Children's Hospital of Soochow University, Laboratory of Aging and Nervous Diseases, Soochow University, Suzhou 215003, China

## Abstract

Epilepsy is the most common childhood neurologic disorder. Status epilepticus (SE), which refers to continuous epileptic seizures, occurs more frequently in children than in adults, and approximately 40–50% of all cases occur in children under 2 years of age. Conventional antiepileptic drugs currently used in clinical practice have a number of adverse side effects. Drug-resistant epilepsy (DRE) can progressively develop in children with persistent SE, necessitating the development of novel therapeutic drugs. During SE, the persistent activation of neurons leads to decreased glutamate clearance with corresponding glutamate accumulation in the synaptic extracellular space, increasing the chance of neuronal excitotoxicity. Our previous study demonstrated that after developmental seizures in rats, E-64d exerts a neuroprotective effect on the seizure-induced brain damage by modulating lipid metabolism enzymes, especially ApoE and ApoJ/clusterin. In this study, we investigated the impact and mechanisms of E-64d administration on neuronal excitotoxicity. To test our hypothesis that E-64d confers neuroprotective effects by regulating autophagy and mitochondrial pathway activity, we simulated neuronal excitotoxicity in vitro using an immortalized hippocampal neuron cell line (HT22). We found that E-64d improved cell viability while reducing oxidative stress and neuronal apoptosis. In addition, E-64d treatment regulated mitochondrial pathway activity and inhibited chaperone-mediated autophagy in HT22 cells. Our findings indicate that E-64d may alleviate glutamate-induced damage via regulation of mitochondrial fission and apoptosis, as well as inhibition of chaperone-mediated autophagy. Thus, E-64d may be a promising therapeutic treatment for hippocampal injury associated with SE.

## 1. Introduction

Epilepsy affects 65 million people worldwide and is a leading neurological cause of loss of quality-adjusted life years [1]. Broadly characterized by aberrant neuronal excitability, epilepsy is the most common neurologic disorder in children, with incidence rates ranging from 33.3 to 82 cases per 100,000 per year [2]. Status epilepticus (SE), or the condition of continuous epileptic seizures, is one of the most common forms of epilepsy. Persistent SE can lead to hippocampal dysfunction and is typified by neurodegeneration, inflammation, altered neurogenesis, and deficits in cognition and memory [3]. SE occurs more frequently in children than in adults, and approximately 40–50% of cases occur in children under 2 years of age [4]. Conventional antiepileptic drugs currently used in clinical practice often have adverse side effects such as headache, drowsiness, nausea, dizziness, and ataxia and are largely unpleasant for patients [5]. Furthermore, persistent SE can lead to the progressive development of drug-resistant epilepsy (DRE), which is often characterized by a resistance to benzodiazepines. The development and progression of DRE is due in part to the N-methyl-D-aspartate receptor (NMDA) receptor-dependent internalization of gamma-aminobutyric acid (GABA) receptors [6]. As current antiepileptic drugs are not able to address this, researchers have increasingly focused on the development of novel therapeutic drugs. Notably, neuroprotection has emerged as a promising therapeutic strategy for preventing and treating epilepsy [7]. To facilitate the application of neuroprotection in therapeutic treatments, a better understanding of the molecular mechanisms that provide neuroprotective effects is required in the context of epilepsy.

Glutamate, a neurotransmitter released by excitatory neurons, plays critical roles in various physiological and pathological brain functions [8, 9]. Glutamate-mediated excitotoxicity has been shown to contribute to the neurobiology of epilepsy. Specifically, it has been found to lead to seizure-induced cell death, increased susceptibility to neuronal synchronization, and network alterations through oxidative stress or excitotoxicity [10–12]. Astrocytes represent the majority of the neuronal cells in the central nervous system. They play a fundamental role in the clearance of neurotransmitters, such as glutamate and GABA [9, 13]. Recent studies have demonstrated that astrocytes may influence the pathogenesis and pathophysiology of epilepsy through the homeostatic control of synaptic transmission via glutamate release [14, 15]. During SE, the persistent neuronal activation of neurons leads to decreased glutamate clearance in the synaptic extracellular space. This results in the accumulation of glutamate, which increases the chance of neuronal excitotoxicity [16]. Previous studies have shown that neuronal cell death plays a major role in epilepsy-related changes in brain function [17, 18]. Further, seizures have increasingly been linked with hippocampal damage and neuronal cell death, usually via apoptosis, and apoptosis has been significantly associated with the activation of the protein Caspase-3 [19–22]. Recent studies have generated novel insights regarding autophagy, which is one of the processes associated with cellular death. Autophagy describes the degradation of a variety of cytoplasmic materials within lysosomes [23]. It has been associated with alterations in Beclin-1, and LC3 protein expression, while necroptosis has been linked with MLKL, and RIP-1 protein expression was associated with necroptosis in four regions (CA1, CA3, DG, and Hilus) of the hippocampus in a rat model of SE. Necroptosis, which is usually after apoptosis, is the promotion of neuronal cell death in response to harmful stimuli such as SE and seizure events. In contrast, autophagy can act as a self-protective mechanism, for instance, by enabling neurons to counteract SE-induced hippocampal neuronal apoptosis [24–27]. Chaperone-mediated autophagy (CMA) plays a fundamental role in the clearance of aggregated proteins and protects against cellular stress and neurodegenerative conditions [28]. This highly selective process of degradation involves cytosolic proteins that are endowed with a Lys-Phe-Glu-Arg-Gln (KFERQ) or KFERQ-like motif in their amino acid sequences. During CMA, a cytosolic chaperone (Hsc70) plays a role in recognizing the KFERQ-like motifs that are present in substrate proteins and controls the subsequent transport of these proteins to Lamp2a (lysosomal-associated membrane protein 2a), which serves as the CMA receptor at the lysosomal surface [9, 28].

Another promising avenue of research is the study of how metabolic dysfunction and homeostatic changes can contribute to seizures and exacerbate related sequelae such as neuronal loss and cognitive impairment [24]. Mitochondria are vital intracellular organelles that undertake many important metabolic roles, and childhood-onset epilepsy is a major phenotypic feature of mitochondrial disorders [29]. Although the mechanisms underlying mitochondrial SE are still not fully understood, recent investigations have revealed that mitochondria can be both the source and the target of metabolic and homeostatic dysfunction during seizures and epilepsy [30]. This may be related to the role of mitochondria producing ATP to maintain Ca^2+^ homeostasis and carry out oxidative phosphorylation. Abnormalities in these processes that are related to insufficient ATP can result in decreased excitability and decreased neuronal survival, as well as the subsequent progression of epilepsy pathogenesis [31]. Although excess Ca^2+^ is another key candidate for these changes, the ways in which changes in intracellular Ca^2+^ stores might influence the features of seizures and epilepsy remain unknown, especially in terms of how excess Ca^2+^ influences activity in the endoplasmic reticulum [32].

Mitochondria are dynamic organelles that continuously undergo fusion and fission, and imbalanced mitochondrial dynamics can lead to distinct neuronal death under pathophysiological conditions [33]. Mitochondrial fission is important in regulating mitochondrial size/shape, as well as in the distribution of mitochondria throughout the cell body, especially in neurons [34]. Dynamin-related protein 1 (Drp1) mediates mitochondrial fission and regulates the mitochondrial fusion-fission balance, which has been associated with neurological disorders such as epilepsy [35, 36]. Mitochondria are also crucial in the initiation of apoptosis. Specifically, mitochondrial outer membrane permeabilization (MOMP) has been shown to regulate apoptosis. Apoptotic signaling downstream of MOMP involves cytochrome c (Cytc), which is released by mitochondria and subsequently activates caspase. Thus, Cytc plays a vital role as a proapoptotic protein [37, 38]. Given the importance of mitochondria in apoptosis, a deeper understanding of the mechanisms underlying mitochondrial changes during SE may facilitate the development of more effective drugs for epilepsy.

**Figure 1:**
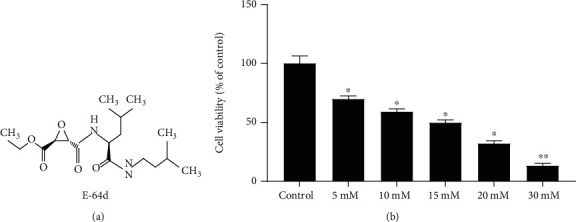
Glutamate induced injuries in HT22 cells. HT22 cells were treated with 5 mM to 30 mM glutamate for 24 hours. (a) Structure of E-64d. (b) Cell viability was evaluated via CCK-8. The data are expressed as the mean ± SD. ^∗^*P* < 0.05 vs. the Control group. ^∗∗^*P* < 0.01 vs. the Control group.

The peptide E-64d is a selective inhibitor of cathepsins B and L (Figure 1(a)) and acts as an autophagy inhibitor. It has previously been shown to be safe as a treatment for Alzheimer's disease in humans and in animal models of focal cerebral ischemia [39, 40]. Previously, our team demonstrated that E-64d exerts a neuroprotective effect in rats. Specifically, E-64d modulated levels of lipid metabolism enzymes, especially ApoE and ApoJ/clusterin, after developmental seizures [41]. However, few studies have used cell models to investigate neuronal excitotoxicity. Based on previous studies, we hypothesized that E-64d may have beneficial effects in models of epilepsy, specifically, by regulating autophagy and the activity of the mitochondrial pathway after neuronal excitotoxicity.

To address this hypothesis in the present study, we used a glutamate-induced excitotoxic model in vitro to investigate whether E-64d could protect against excitotoxicity-induced neuronal injury via the regulation of CMA and activity of the mitochondrial pathway. Furthermore, we sought to better understand the potential protective mechanisms of E-64d against glutamate-induced neuronal excitotoxicity.

## 2. Materials and Methods

### 2.1. Primary Astrocyte Cultures

We prepared primary mouse astrocytes from postnatal 1- or 2-day-old C57BL/6 mice and maintained them in Dulbecco's modified Eagle's medium (DMEM) containing 10% fetal bovine serum (FBS), 100 units/ml penicillin, 100 *μ*g/ml streptomycin, and 2 mM L-glutamine based on previous studies [42, 43]. The experimental protocols were approved by the Experimental Animal Research Ethics Committee of Jiangnan University. Astrocyte purity was verified via immunofluorescence staining with rabbit anti-glial fibrillary acidic protein (GFAP) (Additional file 1: Figure S1). The astrocytes were incubated at 37°C under 5% CO_2_. The medium was changed every 3–4 days, and the astrocytes were used on days 10-12 in vitro according to a previous study [44].

### 2.2. HT22 Cell Lines

We purchased HT22 mouse hippocampal neuronal cells from Guangzhou Jennio Biotech Co., Ltd. (Guangzhou, China).

### 2.3. Reagents and Antibodies

The DMEM, FBS, and penicillin-streptomycin were obtained from Gibco (Grand Island, NY, USA). Trypsin-EDTA was purchased from Absin (Shanghai, China). L-Glutamic acid (glutamate) and dimethyl sulfoxide (DMSO) were sourced from Beijing Solarbio Science & Technology Co., Ltd. (Beijing, China). The (2S,3S)-trans-epoxysuccinyl-L-leucylamido-3-methylbutane ethyl ester (E-64d) was purchased from Sigma-Aldrich Corp. (St. Louis, MO, USA). The Annexin V-FITC/PI apoptosis detection kit was obtained from KeyGEN BioTECH (Nanjing, China). A mitochondrial membrane potential assay kit with JC-1 and the Cell Counting Kit-8 (CCK-8) were purchased from Beyotime Biotechnology (Shanghai, China). The ELISA kit for 8-hydroxydeoxyguanosine (8-OHdG) was supplied by Elabscience Biotechnology Co., Ltd. (Wuhan, China). We purchased antibodies against LC3-II/I, Beclin-1, *β*-Actin, and Caspase-3 from Cell Signaling Technology (Danvers, MA, USA). Antibodies against Hsc70, Drp1, and Cytc were purchased from Abcam (Cambridge, MA, USA).

### 2.4. Cell Culture and Glutamate-Induced Excitotoxicity

Mouse hippocampal neuronal cells (HT22 cells) were cultured in DMEM supplemented with 10% fetal bovine serum, 100 units of penicillin, and 100 *μ*g/ml streptomycin. Before the experiment, the HT22 cells were seeded into 96-well microplates (1 × 10^5^) and incubated at 37°C under 5% CO_2_ for 24 h. To establish the cell model of glutamate-induced excitotoxicity (approximately IC50), we measured the dose response (for 5, 10, 15, and 30 mM glutamate) at 24 h using the CCK-8.

### 2.5. Treatment and Grouping

Cells (1 × 10^5^) were seeded into 6-well or 96-well microplates and incubated for 24 h before the experiment. The control (Control), E-64d alone (E-64d), glutamate injury (Glutamate), and E-64d treatment groups (Glutamate+E-64d) were established as follows. In the E-64d group, E-64d was added to the culture medium at a final concentration of 25 mM based on our previous study [41, 45]. In the Glutamate group, glutamate was added to the culture medium to obtain a final concentration of 15 mM in HT22 cells and 100 mM in primary astrocytes (approximately IC50) [46]. Cells in the Glutamate+E-64d group were simultaneously treated with glutamate and E-64d.

### 2.6. Cell Viability Assay

To examine the viability of the HT22 cells and primary astrocyte cultures, the CCK-8 was used according to previous studies [46, 47]. Briefly, cells were seeded at a density of 5 × 10^3^ cells/well in 96-well tissue culture plates. After 24 h of glutamate stimulation, we exchanged the medium in each well with 100 *μ*l DMEM containing 10 *μ*l CCK-8 solution and then incubated the samples for 1 h at 37°C and 5% CO_2_. Finally, we measured cell viability using a microplate reader with a wavelength of 450 nm. Cell viability was calculated by comparing the experimental groups with the Control group. This experiment was repeated three times.

### 2.7. Apoptosis Assay

To explore alterations in apoptosis, we used Annexin V-fluorescein isothiocyanate (FITC)/propidium iodide (PI) staining according to a previous study [48]. HT22 cells were plated into 24-well plates at a density of 100,000 cells per well. After preincubation and stimulation, the HT22 cells were collected via trypsinization and washed twice with PBS. After being washed, the HT22 cells were resuspended in binding buffer and stained with 5 *μ*l Annexin V-FITC/PI. Then, the cells were incubated in a dark environment for 10 min at room temperature according to the manufacturer's instructions. Finally, the apoptotic ratio was determined via flow cytometry.

### 2.8. Flow Cytometric Detection of Mitochondrial Membrane Potential (ΔΨm)

To measure the mitochondrial membrane potential (MMP) of the HT22 cells, we used flow cytometry according to previous publications [45, 49, 50]. The cells were treated and stained with the mitochondrial membrane potential-sensitive probe JC-1 (Beyotime, Shanghai, China). The cells were then incubated for 30 min with a final concentration of 2 *μ*M JC-1 and washed twice with PBS. For the MMP evaluation, the detection of JC-1 monomers, which aggregate to emit green fluorescence, was conducted according to excitation and emission wavelengths of 485 nm and 535 nm, respectively (FL-1 channel), and the detection of J-aggregates, which aggregate to emit red fluorescence, was conducted with excitation and emission wavelengths of 550 nm and 600 nm, respectively (FL-2 channel). The data were analyzed using FlowJo analysis software, and the results were measured using the ratio of the shift from red to green fluorescence, which reflects changes in the mitochondrial membrane potential [51–53].

### 2.9. 8-Hydroxy-2′-Deoxyguanosine (8-OHdG) Analysis

We measured the level of 8-hydroxy-2′-deoxyguanosine (8-OHdG) as a marker of DNA oxidative damage. We used a commercially available ELISA kit from Elabscience Biotechnology (Wuhan, China). The assays were performed according to the manufacturer's instructions, and the results are given in terms of the level of 8-OHdG.

### 2.10. Western Blot Analysis

To monitor alterations in protein levels, we conducted Western blotting according to previous descriptions [45, 48]. The total proteins from HT22 cells were extracted using radioimmunoprecipitation lysis and extraction buffer (Beyotime Biotechnology) in the presence of protease inhibitors (Roche Applied Science, Indianapolis). Protein concentrations were measured by BCA protein assay (Pierce, Appleton, WI, USA). After separating the proteins via sodium dodecyl sulfate-polyacrylamide gel electrophoresis on a Bis-Tris gel system (Bio-Rad, Hercules, CA), they were then transferred to polyvinylidene fluoride membranes. The membranes were blocked in saline tween (TBST) buffer with 5% nonfat milk for 1 h, then incubated with the following primary antibodies: LC3-II/I (rabbit, 14-16 kDa, 4180S, 1 : 1000 dilution, CST), Beclin-1 (rabbit, 60 kDa, 3738S, 1 : 1000 dilution, CST), Caspase-3 (rabbit, 14-35 kDa, 9662S, 1 : 1000 dilution, CST), *β*-Actin (rabbit, 45 kDa, 4967S, 1 : 1000 dilution, CST), Hsc70 (rabbit, 71 kDa, ab112549, 1 : 5000 dilution, Abcam), Cytc (rabbit, 10-15 kDa, ab133504, 1 : 5000 dilution, Abcam), Drp1 (rabbit, 78-82 kDa, 8570S, 1 : 1000 dilution, CST), and Lamp2a (rabbit, 120 kDa, ab125068, 1 : 5000 dilution, Abcam) overnight at 4°C. On the next day, the membranes were washed and incubated with an HRP-conjugated secondary antibody for 1 h at room temperature. The proteins were imaged using chemiluminescent autography. The optical density of the image was measured and analyzed using image analysis software.

### 2.11. Immunocytochemistry

To investigate possible changes in Cytc, Drp1, Hsc70, and Lamp2a in the context of glutamate-induced neuronal excitotoxicity, we performed immunocytochemistry according to a previous study [54]. Cells (1 × 10^5^) were seeded into 24-well microplates and fixed with 4% paraformaldehyde in PBS. The cells were permeabilized with 0.1% Triton X-100 in PBS (PBST) for 15 min and treated with 5% bovine serum albumin in 0.25% PBST. The cells were then incubated overnight at 4°C with the anti-LC3, anti-Cytc, anti-Drp1, anti-Lamp2a, and anti-Hsc70 antibodies diluted in blocking solution. Next, the cells were incubated with Alexa 488 goat anti-rabbit secondary antibodies (Thermo Fisher Scientific) overnight at 4°C and washed with PBS. The microplates were mounted on confocal slides with the VECTASHIELD mounting medium. Images were obtained using an LSM-800 confocal microscope (Carl Zeiss). Confocal analysis was performed in triplicate.

### 2.12. Statistical Analysis

All experiments were carried out at least three times, and all statistical analyses were performed using SPSS 19.0 software (IBM Corporation, Armonk). The results are expressed as the mean ± standard deviation of three independent experiments. Differences were evaluated using Student's *t*-test and one-way analysis of variance followed by Dunnett's *t*-test. *P* < 0.05 was considered to indicate statistical significance. All statistical tests were performed using GraphPad Prism Version 6.0 (GraphPad Prism Software, Inc., CA, USA).

## 3. Results

### 3.1. E-64d Protects Cells against Glutamate-Induced Neuroexcitotoxicity

Based on the relevant literature [50, 55] and our preliminary experiments, we first evaluated the dose-dependent responses of HT22 cells to glutamate using the CCK-8. Then, we examined changes in primary astrocytes to confirm the protective effect of E-64d against glutamate-induced neuroexcitotoxicity based on a previous study [46]. Our findings confirmed the efficacy of the experimental model of neuroexcitotoxicity in HT22 cells. Specifically, we found that 15 mM of glutamate induced cell death by approximately 50% after 24 h (*P* < 0.01, Figure 1(b)).

**Figure 2:**
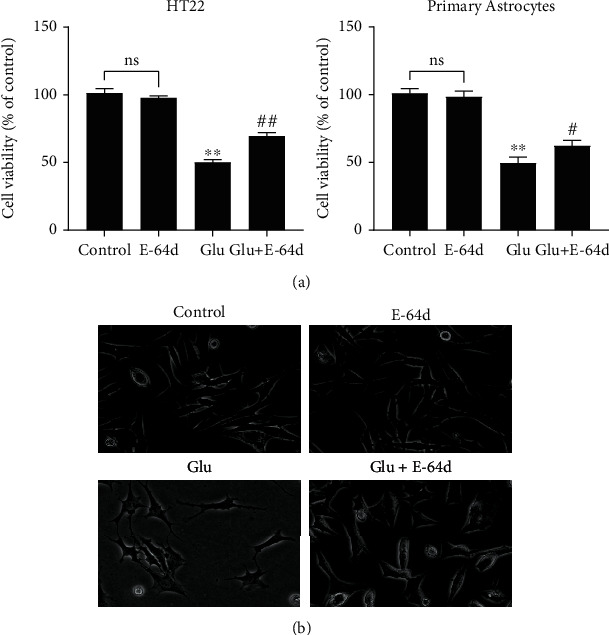
Protective effect of E-64d on glutamate-induced injury in HT22 cells and primary astrocytes. (a) Cell viability was evaluated via the CCK-8. (b) Observation of HT22 cell morphology in each group via light microscopy (×10). The data are expressed as the mean ± SD. ^∗∗^*P* < 0.01 vs. the Control group. ^#^*P* < 0.05, ^##^*P* < 0.01 vs. the Glutamate group. n.s.: not significant.

When E-64d was applied at a concentration of 25 *μ*M, it did not affect the viability of HT22 cells or primary astrocytes, and treatment with E-64d significantly prevented the reduction in cell viability compared with glutamate treatment (*P* < 0.05, Figure 2(a)).

Additionally, light microscopy revealed that cells in the glutamate injury group exhibited weak adherence and smaller cell volume, but after E-64d intervention, the cell morphology tended to be normal (Figure 2(b)). These results suggest that E-64d protected HT22 cells and primary astrocytes against glutamate-induced neuroexcitotoxicity.

### 3.2. E-64d Alleviates Glutamate-Induced Apoptosis

**Figure 3:**
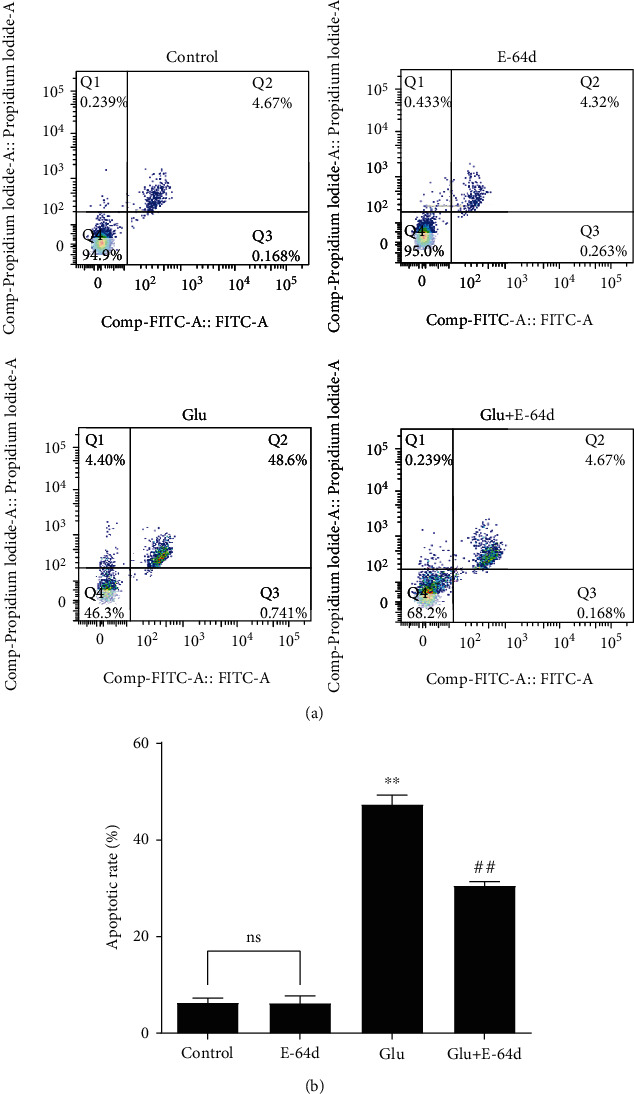
Apoptosis rates of glutamate-treated HT22 cells following 24 h of exposure to E-64d. (a) The cell apoptosis rate was measured via flow cytometry. (b) Quantitative analysis of the apoptosis rate in cells. Three independent experiments were performed, and the data are expressed as the mean ± SD. ^∗∗^*P* < 0.01 vs. the Control group. ^##^*P* < 0.01 vs. the Glutamate group. n.s.: not significant.

The hippocampus is highly vulnerable to epileptiform activity, which is associated with neuronal apoptosis [56]. To further investigate the protective effect of E-64d on hippocampal excitotoxic neuronal injury, we examined the apoptosis rate. As shown in Figures 3(a) and 3(b), we found that the apoptosis rate increased sharply after exposure to glutamate (*P* < 0.01), whereas treatment with E-64d alleviated glutamate-induced apoptosis. Taken together, these results demonstrate that E-64d can alleviate glutamate-induced apoptosis.

### 3.3. E-64d Reverses the Decrease in Glutamate-Induced Mitochondrial Membrane Potential

**Figure 4:**
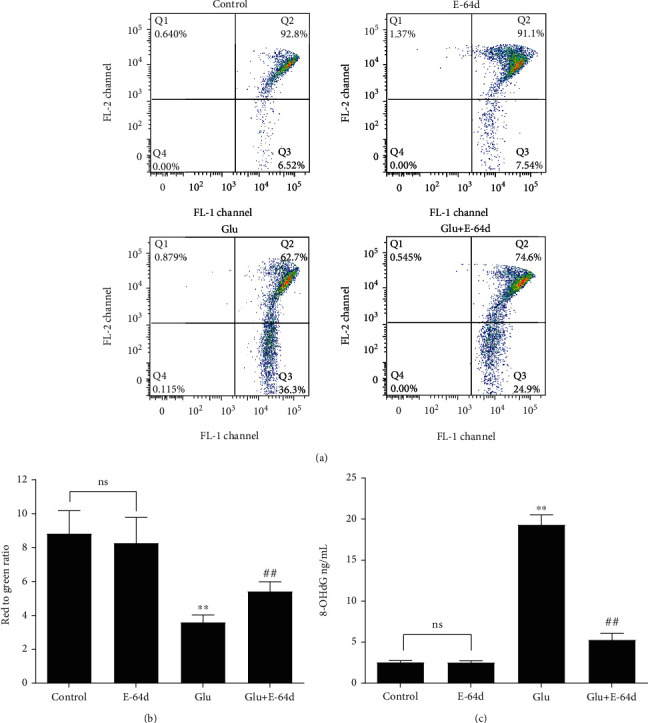
Effect of E-64d on glutamate-induced changes in mitochondrial membrane potential in terms of JC-1 activity and oxidative stress, as detected via ELISA. (a) Measurement of mitochondrial membrane potential via flow cytometry. (b) Quantitative analysis of mitochondrial membrane potential in HT22 cells. (c) Quantitative analyses of the average levels of 8-OHdG in cells. Three independent experiments were performed, and the data are expressed as the mean ± SD. ^∗∗^*P* < 0.01 vs. the Control group. ^##^*P* < 0.01 vs. the Glutamate group. n.s.: not significant.

JC-1 forms a polymer in the normal mitochondrial matrix which is labeled via red fluorescence. When a mitochondrion depolarizes, JC-1 effluxes to the cytoplasm, where it then emits green fluorescence. A decrease in the mitochondrial membrane potential is a marker event of the early stage of apoptosis [57]. Therefore, we used a change in JC-1 from red to green fluorescence as an indicator of early-stage apoptosis [51–53]. The flow cytometry results showed there were more green cells in the Glutamate group, which suggests that the mitochondrial membrane potential had decreased. In contrast, mitochondrial membrane potential increased in the Glutamate+E-64d group (Figures 4(a) and 4(b)). These results indicate that E-64d reversed the glutamate-induced decrease in mitochondrial membrane potential and inhibited apoptosis.

### 3.4. E-64d Alleviates Glutamate-Induced Oxidative Stress in Hippocampal Cells

Oxidative stress has been identified as an intrinsic mechanism for the initiation and progression of epilepsy [58, 59]. In nuclear and mitochondrial DNA, 8-hydroxy-2′-deoxyguanosine (8-OHdG) is an indicator of oxidative DNA damage and has therefore been widely used as a biomarker of oxidative stress [60, 61]. As shown in Figure 4(c), we observed significant increases in 8-OHdG levels in the Glutamate group compared to the Control group. However, these changes were reversed by E-64d administration. These results indicate that E-64d significantly affects oxidative damage caused by glutamate.

### 3.5. E-64d Inhibits CMA and Regulates Mitochondrial Pathway Activity

**Figure 5:**
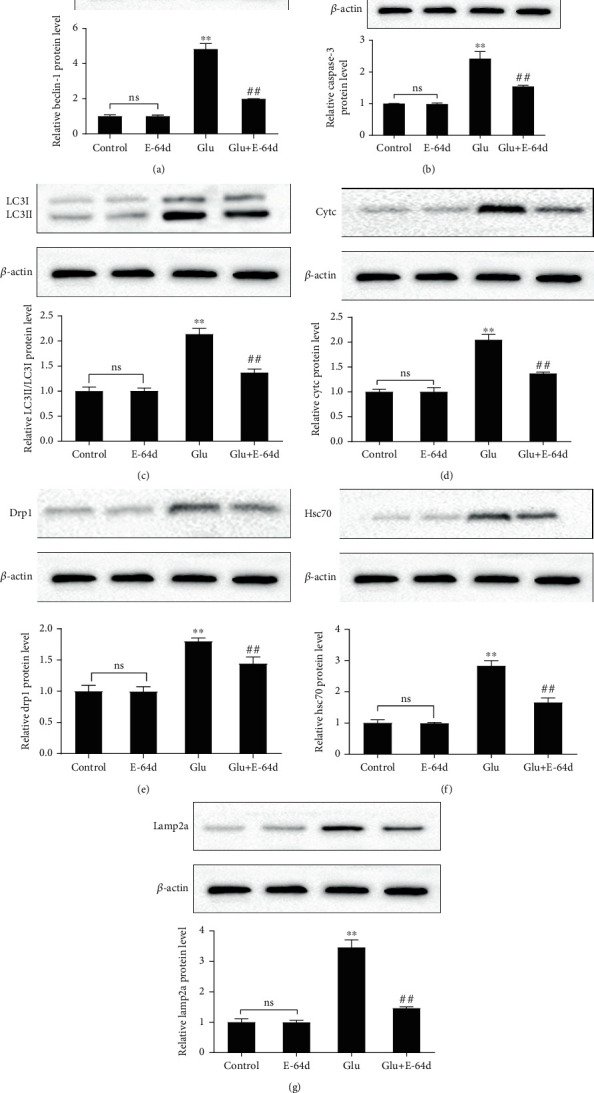
HT22 cells were treated with glutamate and E-64d for 24 h. The protein expression of Beclin-1 (a), Caspase-3 (b), LC3-II/I (c), Cytc (d), Drp1 (e), Hsc70 (f), and Lamp2a (g) was measured by Western blotting. Three independent experiments were performed, and the data are expressed as the mean ± SD. ^∗∗^*P* < 0.01 vs. the Control group. ^##^*P* < 0.01 vs. the Glutamate group. n.s.: not significant.

**Figure 6:**
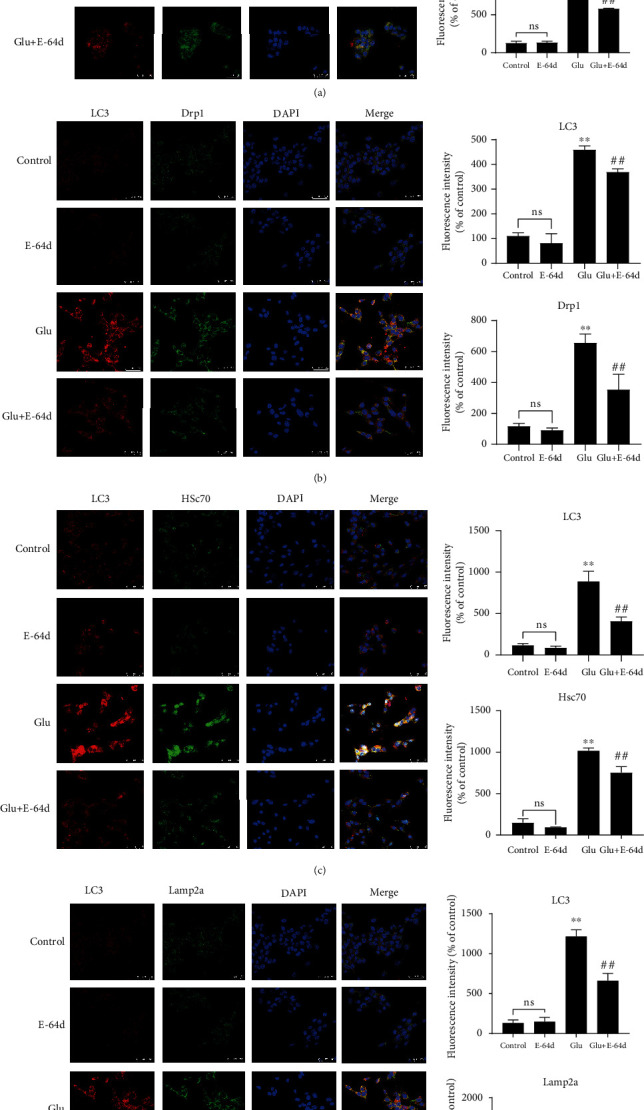
Effect of E-64d on protein expression of Cytc (a), Drp1 (b), Hsc70 (c), and Lamp2a (d) in HT22 cells, as determined by laser confocal microscopy. The four groups were stained with anti-LC3 antibodies (red); anti-Cytc, Drp1, Hsc70, and Lamp2a antibodies (green); and DAPI (blue, cell nuclei labeling). The results of the quantitative analysis of average fluorescence intensity are shown. ^∗∗^*P* < 0.01 vs. the Control group. ^##^*P* < 0.01 vs. the Glutamate group. n.s.: not significant.

We determined the effects of E-64d on the expression of CMA, mitochondrial fission, and apoptosis-related proteins using Western blotting. The results showed a large increase in the expression of Beclin-1, Caspase-3, Cytc, Hsc70, Drp1, Lamp2a, and LC3-II/I in the Glutamate group compared to the Control group; however, E-64d administration reversed these effects (Figure 5). To further confirm the possible protective effects of E-64d on glutamate-induced neuronal excitotoxicity, we performed confocal immunofluorescence analysis and found that E-64d treatment led to a significant decrease in the number of LC3 puncta, Cytc, Hsc70, Drp1, and Lamp2a (Figure 6). Taken together, these results indicate that E-64d protects HT22 cells by inhibiting CMA, regulating mitochondrial fission, and modulating apoptosis pathways.

## 4. Discussion

In this study, we further investigated the potential mechanisms of E-64d treatment in HT22 cells after glutamate-induced damage. Our results indicated that E-64d protected HT22 cells against glutamate-induced injury. To evaluate the effects of E-64d on neuronal cells, we first established an in vitro model by exposing HT22 cells and primary mouse astrocytes to 15 mM glutamate for 24 h. Our findings are consistent with our previous work, suggesting that E-64d exerts a neuroprotective effect against epilepsy-induced neuronal damage [41, 62]. Using this glutamate-induced injury model, we then investigated the mechanisms by which E-64d exerts protective effects. We focused on alterations in mitochondrial fission, apoptosis, and CMA, which have been associated with the modulation of cell death in response to hippocampal injury [34, 53, 63, 64]. Epilepsy is frequently seen in mitochondrial disease and has been reported in >20% of adult cases and 40%–60% of pediatric cohorts [65]. Mitochondrial fission is needed for mitochondrial motility in the G2/M phase of the cell cycle and plays an important role in the distribution of mitochondria across the neuronal cell body [34]. Although the relationship between SE and mitochondrial fission has not been comprehensively examined, several abnormalities in mitochondrial dynamics have been linked to SE, including mitochondrial fission protein Drp1 [65]. In addition, apoptosis is the most common pathological feature associated with neurodegenerative diseases, and apoptosis is known to be regulated by mitochondria. In epilepsy, mitochondrial impairments lead to decreased energy and abnormal reactive oxygen species (ROS) production, which initiate apoptotic neuronal death [66]. A number of studies have indicated that mitochondrial apoptosis might be a protective mechanism in epilepsy [33, 67–69]. To the best of our knowledge, this study is the first to demonstrate that E-64d prevents glutamate-induced hippocampal cell injury via the mitochondrial fission and apoptosis pathways.

At present, the best-characterized form of autophagy is macroautophagy, which is a process in which cargo that is sequestered in double-membrane vesicles (autophagosomes) is delivered to lysosomes through vesicular fusion [70]. In contrast, CMA is a selective type of autophagy in which a specific subset of intracellular proteins is targeted by the lysosome for degradation [71]. Despite large advances in the theoretical understanding of CMA during the past several decades, CMA is still not fully understood [70]. Previous studies have shown that CMA plays an important role in Parkinson's disease, Alzheimer's disease, and cancer [72–74]; however, whether CMA is induced during SE is not clear. In the present study, we newly demonstrated for the first time that E-64d prevented glutamate-induced hippocampal cell injury via the CMA pathway.

## 5. Conclusions

Collectively, our data indicate that E-64d alleviates glutamate-induced damage by regulating mitochondrial fission and apoptosis, as well as inhibiting CMA in HT22 cells. Accordingly, E-64d may be a promising therapeutic candidate for hippocampal injury associated with SE. However, additional in-depth studies are required to fully assess the role of E-64d in epilepsy.

## Data Availability

The data used to support the findings of this study are available from the corresponding authors upon request.

## Supplementary Materials

Supplementary Materials Additional file 1: primary astrocytes were purified and verified via immunofluorescence for glial fibrillary acidic protein (GFAP).
