# Supplementary figures and images for: The Protective Role of E-64d in Hippocampal Excitotoxic Neuronal Injury Induced by Glutamate in HT22 Hippocampal Neuronal Cells
Source: Neural Plast. 2021 Oct 20;2021:7174287. doi: 10.1155/2021/7174287 (PMC8550833; doi:10.1155/2021/7174287)

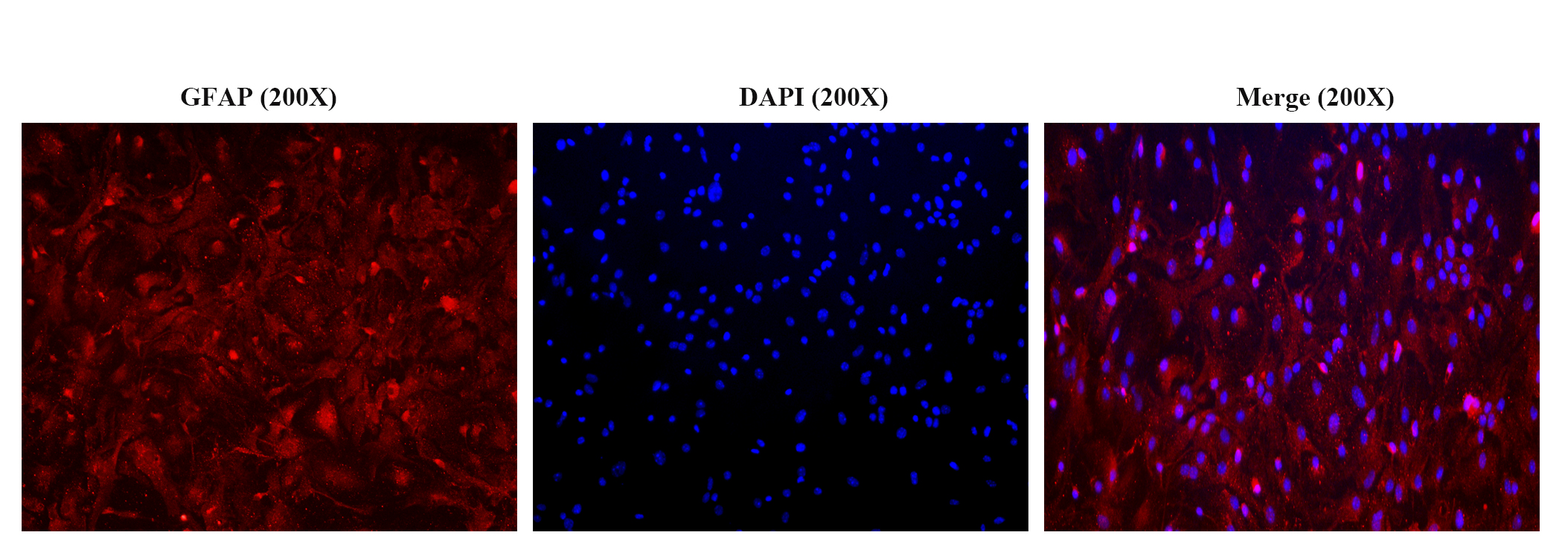

Supplement: Supplementary Materials — Additional file 1: primary astrocytes were purified and verified via immunofluorescence for glial fibrillary acidic protein (GFAP). [file 7174287.f1.jpg]
